# AssayBLAST v2: major update improving reliability and reporting of the *in silico* analysis of molecular multi-parameter assays

**DOI:** 10.1186/s12859-026-06595-w

**Published:** 2026-08-31

**Authors:** Tom Eulenfeld, Maximillian Collatz, Sascha D. Braun, Ralf Ehricht

**Affiliations:** 1 RNA Bioinformatics and High-Throughput Analysis, Friedrich Schiller University Jena, Jena, Germany; 2 Bioinformatics Core Facility Jena, Friedrich Schiller University Jena, Jena, Germany; 3 Leibniz Institute of Photonic Technology, Member of the Research Alliance ”Leibniz Health Technologies” and the Leibniz Centre for Photonics in Infection Research (LPI), 07745 Jena, Germany; 4 InfectoGnostics Research Campus Jena, Center for Applied Research, 07743 Jena, Germany; 5 Center for Translational Medicine (CETRAMED), Jena University Hospital, Friedrich Schiller University Jena, 07747 Jena, Germany; 6 Institute of Physical Chemistry, Friedrich Schiller University Jena, 07743 Jena, Germany

**Keywords:** Oligonucleotide design, Molecular multi-parameter assay, PCR

## Abstract

**Introduction:**

Accurate *in silico* evaluation of primers and probes is essential for the rational design of molecular multi-parameter assays. We present AssayBLAST v2 to automate and simplify this process for extensive assay designs.

**Results:**

A newly integrated strand and proximity check enables precise validation of corresponding oligonucleotides, ensuring correct orientation and spacing required for amplification. Based on predicted oligonucleotide interactions, AssayBLAST v2 determines the theoretical amplification outcomes, offering a computational benchmark for downstream wet-lab validation and performance correlation. Additionally, the updated software integrates an adaptive BLAST parameter optimization that dynamically scales with database size, thereby improving both analytical sensitivity and computational performance. These improvements are supported by a comparative evaluation against the previous version of AssayBLAST.

**Conclusions:**

Collectively, these enhancements streamline the assay development workflow, reduce costs associated with suboptimal primer and probe synthesis, and increase the robustness and reliability of molecular diagnostics and research applications.

## Background

Molecular methods such as PCR, qPCR, isothermal amplification, and DNA microarrays rely on the specific binding of short oligonucleotides to their preferably unique complementary counterpart [9, 11, 12]. Therefore, the design of molecular multi-parameter assays critically depends on accurate in silico evaluation of primers and probes, a task that is often complex and nontrivial. With the release of AssayBLAST, users gained not only a comprehensive assessment of individual oligonucleotides’ performance but also the ability to detect unexpected off-target binding events early in the design process, thereby reducing the risk of unnecessary and costly wet-lab experimentation [4]. Importantly, AssayBLAST allows users to build a custom BLAST database [2, Basic Local Alignment Search Tool] composed of intended target sequences and additional genomes or sequence sets representing potential off-target or background sequences. This flexibility enables the user to refine oligonucleotide designs even for assays requiring extreme specificity, such as those employed in bacterial outbreak screening or clinical diagnostics. Because AssayBLAST specificity predictions are highly accurate in practice, for example, achieving a 97.5% concordance with experimental microarray hybridization results in the original publication Collatz et al. [4], the output provides a solid foundation for optimizing downstream wet-lab protocols to reflect predicted binding behavior.

Version 2 of AssayBLAST was rewritten entirely from scratch. Significant enhancements include automatic assignment and classification of nearby oligonucleotides, as well as improved presentation of results. In addition, key BLAST parameters are now dynamically optimized, which improves the tool’s runtime and reliability. These and further modifications are described in detail in the following section. Evaluations against the previous version of AssayBLAST are presented in Sect. 3.

## Implementation

### Automatic adjustment of key BLAST parameters

In AssayBLAST, users define match filtering by specifying the number of allowed mismatches (parameter --mismatch). BLAST does not allow the direct use of this parameter. Instead, an *E-value* threshold is used. The E-value of a hit is the expected number of random matches with the same or better score found in a database of the same size. It is a critical parameter because BLAST applies this threshold during an early filtering step. Lower E-value thresholds generally reduce runtime by excluding low-scoring matches. However, if the threshold is set too low, valid hits that satisfy the user-defined mismatch criteria may be missed. In previous versions of AssayBLAST, the E-value threshold was hard-coded to 1000 [4]. With this setting, valid BLAST hits were frequently missed when screening larger databases. For instance, for databases larger than 300 MB, hits with two or more mismatches are missed for a query length of 18 nucleotides. To mitigate this issue, the threshold was increased to 100,000 in AssayBLAST v1. This value was selected empirically and represented a conservative heuristic intended to maximize sensitivity across a wide range of database sizes. However, this approach also increased the number of candidate hits evaluated by BLAST, resulting in unnecessarily long runtimes for many applications. In version 2, an appropriate E-value threshold is automatically calculated based on the database size and query length to maximize sensitivity while maintaining efficient search performance. In a first step, the alignment score *S* for a hit of the shortest query with the maximum number of allowed mismatches is calculated with

1 $$\begin{aligned} S = 5(Q-M) - 4M \,. \end{aligned}$$

**Fig. 1:**
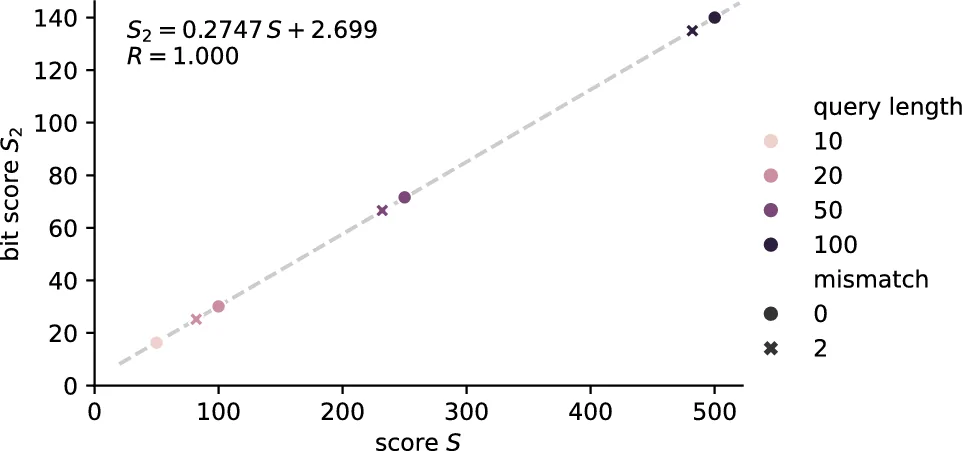
Relationship between BLAST bit score and raw alignment score. The mapping depends on the underlying scoring system, which is fixed in AssayBLAST

Here, *Q* denotes the length of the shortest query sequence and *M* the number of allowed mismatches (parameter --mismatch, default is 2). The reward for a match is 5, the penalty for a mismatch is 4. As gaps in sequence alignments do not occur in biological reality but are merely artifacts of sequence comparison algorithms, we assigned a high gap cost of 1000 to prevent their inclusion in BLAST alignments. The bit score $S_2$, the $\log _2$-scaled required size of a database to get a match by chance, is calculated from the score *S*. The linear relationship between $S_2$ and *S* depends on the scoring system and the derived parameters of the Gumbel distribution, which are calculated within BLAST [1, 2, 10]. We approximate this relationship by performing a simple linear regression between the calculated scores *S* and BLAST-reported bit scores $S_2$, based on hits spanning different query lengths and numbers of mismatches (see Fig. 1):

2 $$\begin{aligned} S_2 = 0.2747\,S + 2.699 \end{aligned}$$

We verified that this relationship is independent of sequence characteristics by evaluating genomes from different organisms. The E-value threshold *E* used in the BLAST call can be calculated from the bit score $S_2$ and the database size *D* with

3 $$\begin{aligned} E = QD / 2^{FS_2}\,. \end{aligned}$$

The safety factor, denoted by $F{=}0.95$, accounts for rounding errors and the internal BLAST recalculation of E-values [8]. Although the calculated E-value threshold guarantees the identification of all matches with the permitted number of nucleotide mismatches or fewer, matches with more mismatches may be included due to queries longer than *Q*. These hits are filtered out later when the BLAST results are analyzed. Finally, the BLAST parameter -max_target_seqs is set to 1,000,000,000 to ensure that no hits are missed. It is in the order of the maximum allowed value for this parameter, a signed long integer ($2^{31}-1$).

### Classification of nearby oligonucleotides and enhanced reporting

Version 1 of AssayBLAST reported the BLAST hits in a table with columns for each oligonucleotide-strand combination and rows for each database sequence. Table 1 reports the results of version 1 using the dataset of Collatz et al. [4] reduced to three sequences. In previous versions, users manually paired probe and primer hits based on positional proximity and strand orientation to infer amplification types.

**Fig. 2:**
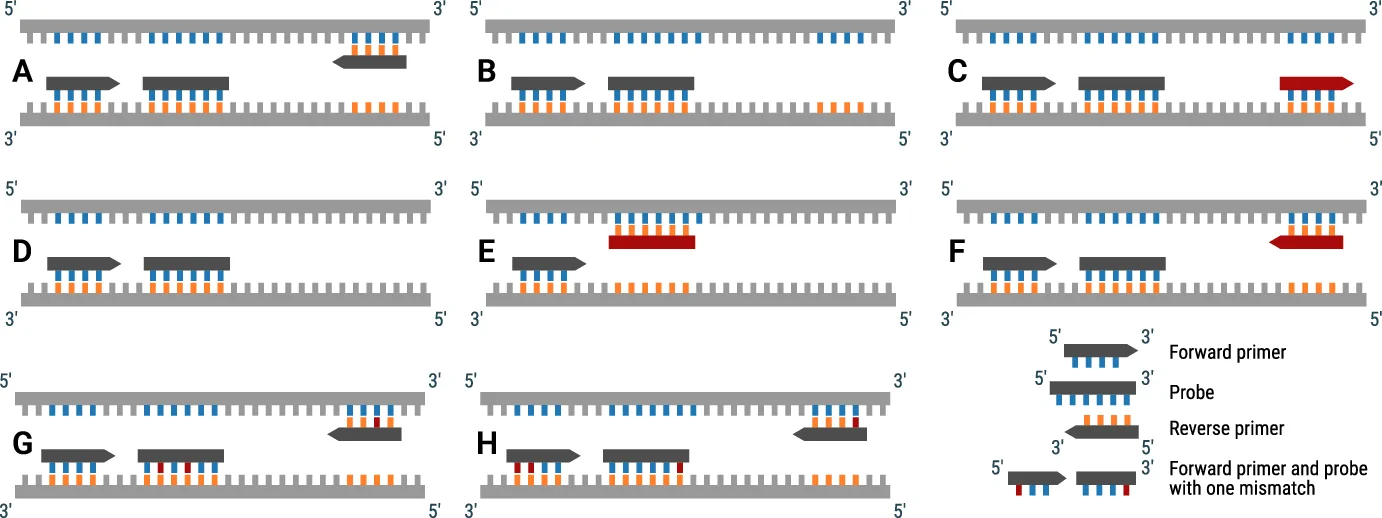
Oligonucleotide interactions identified by AssayBLAST version 2. Top row: A user-designed interaction with exponential amplification is identified in example A. In example B, the reverse primer is missing or binds outside of the specified distance. In example C, the reverse primer binds to the wrong strand. For both examples B and C a linear amplification is reported by AssayBLAST. Middle row: A user-designed interaction with linear amplification is identified in example D. In example E, the probe binds to the wrong strand, leading to no amplification. In example F, an additional primer—unintended by the user—binds to the forward strand, resulting in unexpected exponential amplification. Bottom row: AssayBLAST also reports the number of mismatches for each oligonucleotide (G), specifically highlighting mismatches at the probe’s 5’ end or the primer’s 3’ end (H), as these negatively affect the transcription

**Table 1:** Obsolete results table for AssayBLAST version 1

	primer_lukF _11b_forward	primer_lukF _11b_revcomp	probe_lukF _10_forward	probe_lukF _10_revcomp	primer_entN _51_forward	primer_entN _51_revcomp	probe_entN _11_forward	probe_entN _11_revcomp
CP102956		0 (pos: 1942449- 1942466)	0 (pos: 1942420- 1942445)					
CP102957	0 (pos: 2204112- 2204129)			0 (pos: 2204133-2204158)				
CP102958	0 (pos: 2344146- 2344163)			0 (pos: 2344167- 2344192)		0 (pos: 184189- 184210)	0 (pos: 184157- 184181)	

Each oligonucleotide-strand combination has its own column. Theoretical amplifications are not inferred. The displayed data is an excerpt of the used data [4]

**Table 2:** The AssayBLAST version 2 overview table indicates the inferred amplification and provides a description of the paired oligonucleotides for each probe and database sequence

Genome	probe_entN_11	probe_lukF_10
CP102956	none	lin P0+ R0−
CP102957	none	lin F0+ P0−
CP102958	lin P0+ R0−	lin F0+ P0−

Table headers correspond to user-defined probe names provided in the input file. Cells use the following abbreviations: F: forward primer; P: probe; R: reverse primer. Further details are provided in the text. The table was generated using the same dataset as in Table 1

**Table 3:** The AssayBLAST version 2 detailed table characterizes all paired oligonucleotide hits

Genome	Probe	Amp	Pairing and strand direction ((+)/(−))	SD	SC	MM	Dist	Location
CP102956	probe_lukF_10	lin	probe_lukF_10 (+),primer_lukF_11b (−)	+ −	Pass	M0,0	D3	1942420-1942445,1942449-1942466
CP102957	probe_lukF_10	lin	primer_lukF_11b (+),probe_lukF_10 (−)	+ −	Pass	M0,0	D3	2204112-2204129,2204133-2204158
CP102958	probe_entN_11	lin	probe_entN_11 (+),primer_entN_51 (−)	+ −	Pass	M0,0	D7	184157-184181,184189-184210
CP102958	probe_lukF_10	lin	primer_lukF_11b (+),probe_lukF_10 (−)	+ −	Pass	M0,0	D3	2344146-2344163,2344167-2344192

Each cell in Table 2 corresponds to a row in Table 3. The abbreviations in the column headers are as follows: Amp: Amplification; SD: Strand direction; SC: Strand check; MM: Mismatch; Dist: Distance. The table was generated using the same dataset as in Table 1

The new version of AssayBLAST performs these tasks automatically and can therefore be used to identify and differentiate designed and unintended oligonucleotide interactions, as exemplified in Fig. 2. For this purpose, the BLAST hits for each searched sequence are first sorted by their genomic position. Then, the tool iterates through all BLAST hits corresponding to probes and searches for primer hits up to a specified nucleotide distance on the 5’ and 3’ sides (--distance option, default is 250 nucleotides). Additionally, the user can switch to a mode that only checks for primer-primer interactions with the --only-primer flag. The results are reported in two tables in tab-separated value (TSV) format. The overview table, see Table 2, reports the inferred amplification for each probe and sequence, along with a string representing the best probe/primer hits. Table headers correspond to user-defined probe names provided in the input file. Valid amplification values are:

- none: no probe found,
- noprimer: probe found, but no primers found,
- lin: linear amplification, a working primer was found on one side of the probe,
- exp: exponential amplification, working primers were found on both sides of the probe.

The oligonucleotide combination pattern follows the regular expression:

(F *\backslash * d[+-])? (P *\backslash * d[+-])? (R *\backslash * d[+-])?

F, P, and R denote the forward primer (located on the 5’ side of the probe), the probe and the reverse primer (located on the 3’ side of the probe), respectively. The number after that denotes the number of mismatches of the hit, and finally, the strand on which the hit is located is indicated by + or-. Note that a “working” primer necessarily requires the forward primer to be located on the forward strand (+) and the reverse primer to be located on the reverse strand (−).

The detailed table, see Table 3, reports a row for each oligonucleotide interaction. Therefore, each cell in Table 2 corresponds to a row in Table 3. Each row states the genome, the probe ID, the expected amplification, and the paired oligonucleotide IDs and strand directions. The next column gives the strand directions of the oligonucleotides. The strand check column displays the result checking whether the primers are located on the correct strand (see above). The mismatch column displays the number of mismatching nucleotides for each hit. A mismatch directly at the 5’ end of the probe or the 3’ end of the primer is indicated by an asterisk, as this negatively influences their interaction. The distance column displays the distance in nucleotides between the hits. The final column shows the exact locations of the BLAST hits.

### Other updates

We now publish AssayBLAST on PyPI, which allows for easy installation. Beginning with version 2, the workflow has been modularized into two command-line utilities. The assay_blast script calculates the BLAST parameters and runs the BLAST search. The results are analyzed with the assay_analyze script. Since the BLAST search is the most time-consuming part of the pipeline, this separation enables reuse of stored BLAST results, allowing downstream analyses with modified parameters without re-executing the BLAST search. Additionally, AssayBLAST version 2 uses a single BLAST call for the forward and reverse strands, rather than two separate calls as in version 1, since BLAST inherently evaluates both strands within a single query. This consolidation reduces redundant computations and improves runtime efficiency. We also introduced a warning for duplicate primer or probe sequences in the query file, including detection of reverse-complement duplicates. Note that this check does not model partial primer-primer interactions. The BLAST output is parsed using the Sugar library [5], which supports the detection and parsing of various feature formats. Consequently, the assay_analyze script can also be used with MMSeqs2 output [13] or GFF/GTF files. The Sugar library is also used for the oligonucleotide matching.

## Results and discussion

**Fig. 3:**
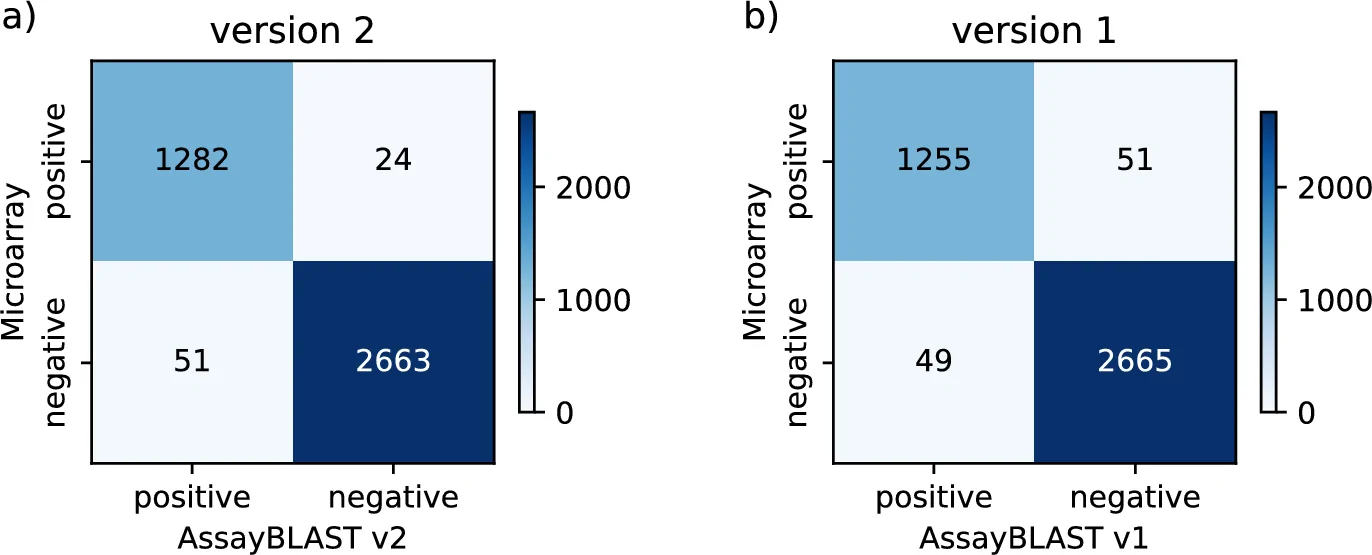
**a** Confusion matrix comparing the automated interpretation of AssayBLAST version 2 results with the DNA microarray results reported in Collatz et al. [4]. **b** Corresponding confusion matrix for AssayBLAST version 1 [4, Fig. 1 therin]. The accuracy of AssayBLAST version 2 is improved compared to the previous version

Experimental validation of AssayBLAST predictions using qPCR assays was previously reported by Collatz et al. [4]. In the present study, we employ the DNA microarray dataset introduced in that work to compare AssayBLAST v2 and v1 under identical conditions. Although DNA microarray hybridization and PCR amplification rely on different biochemical mechanisms, both are governed by sequence-specific oligonucleotide-target interactions. Consequently, the microarray dataset provides a suitable benchmark for evaluating the sequence-based specificity prediction logic implemented in AssayBLAST and for quantifying the impact of the algorithmic improvements introduced in version 2. We use the same threshold of 0.5 to classify microarray experiments as positive or negative. The confusion matrix comparing the automated interpretation of AssayBLAST version 2 results versus the DNA microarray survey is displayed in Fig. 3a. The false negative rate is reduced by more than 50 % compared to AssayBLAST version 1 (Fig. 3b), resulting in an increase in accuracy from 97.5 % to 98.1 %. The improved results may be explained by AssayBLAST v1 missing some valid interactions, as discussed below, due to missed hits with mismatches near query ends.

We also compare the correctness and runtime of AssayBLAST v2 with the previous version. For this purpose, we downloaded complete genomes from diverse organisms: *Influenzavirus A* (H3N2 subtype, GCA_039834415.1), *Staphylococcus aureus* (GCF_000568455.1), *Candida albicans* (GCF_000182965.3), and *Homo sapiens* (T2T, GCF_009914755.1). We generated queries of lengths 10 nt, 20 nt, 50 nt, and 100 nt by extracting sequences of the corresponding lengths from the beginning of each genome. We then evaluated different versions of AssayBLAST to identify query hits within the genomic regions. For AssayBLAST version 1, we considered two configurations: one with a fixed E-value threshold of *1 000*, as reported in Collatz et al. [4], and one with a fixed threshold of *100,000*, corresponding to the version tagged on GitHub. For the evaluation we use a single core of an AMD EPYC 9754 128-Core Zen 2.25 GHz machine. Correctness of results and run times are reported in Table 4. A detailed report of BLAST hits for a single row of Table 4 is provided in Table 5. Two issues were identified with AssayBLAST v1. First, version 1 could fail to identify interactions when the fixed E-value threshold was lower than or comparable to the automatically calculated E-value threshold used in version 2. This mainly affects combinations of large genomes and short queries, particularly when mismatches are allowed (e.g., Table 4, *Candida albicans*, 10 nt). Consequently, cases in which version 1 reports fewer hits than version 2 are considered incorrect if the E-value threshold determined by version 2 exceeds the fixed threshold for version 1. Second, when mismatches are allowed, AssayBLAST v1 inadvertently permits gaps during BLAST alignment. As a result, erroneous interactions are reported in version 1 (e.g., *Homo sapiens*, 50 nt). In addition, version 1 fails to report interactions containing mismatches at the ends of the query sequence (e.g., *Candida albicans*, 20 nt and Table 5). Consequently, cases in which version 1 reports a different number of hits than version 2 are considered incorrect when mismatches are permitted. The example shown in Table 5 represents another incorrect result of version 1, although the total hit counts are identical. When the fixed E-value threshold in version 1 is substantially higher than the automatically calculated E-value threshold, AssayBLAST v2 achieves improved runtime due to increased BLAST search efficiency. The largest speedup was observed for long genomes and long queries (e.g., *Homo sapiens*, 100 nt).

**Table 4:**
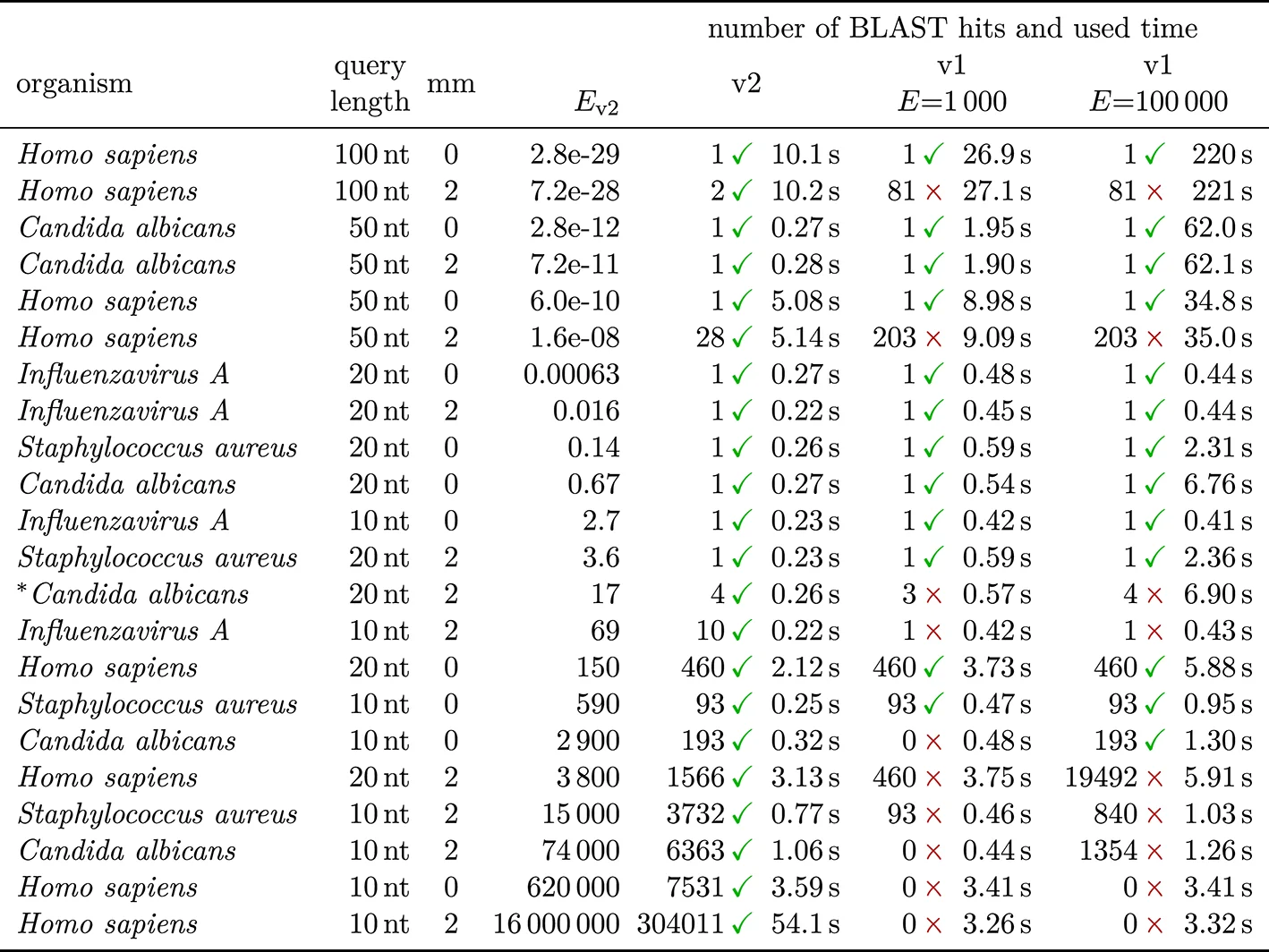
Evaluation of AssayBLAST v2 compared to AssayBLAST v1

The number of BLAST hits is reported for different organisms, query lengths, and allowed mismatch counts (column mm), along with whether AssayBLAST v1 correctly identified these hits and the corresponding runtime. The automatically determined E-value threshold (column $E_{\textrm{v2}}$) improves both analytical sensitivity and computational performance. The table is sorted by this column. Details about the BLAST hits for the row marked with an asterisk are given in Table 5

**Table 5:**
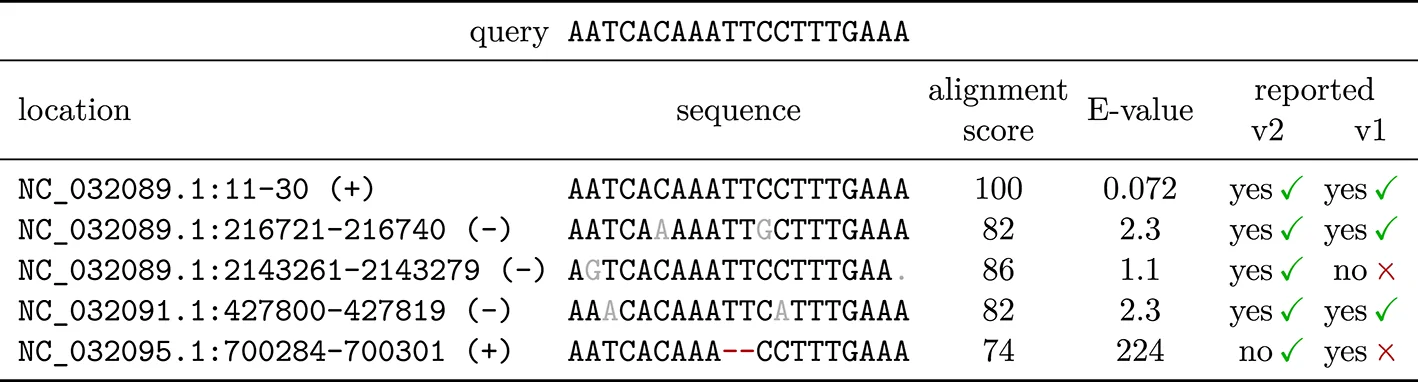
Hits reported by AssayBLAST v2 and v1 for the 20 nt query in *Candida albicans* with a mismatch parameter of 2

The table indicates whether each genomic location was reported by version 2 or version 1 and whether the corresponding report is considered correct. E-values are reported for version 2 only, except for locations not identified by version 2, where the E-value from version 1 is shown instead. Note that E-values from version 1 and version 2 are not directly comparable, because version 1 uses a different scoring system that permits gaps. The table corresponds to the row *Candida albicans*, 20 nt, mm 2 marked with an asterisk in Table 4. Version 1 reports hits containing gaps, which do not permit a valid interaction and therefore should not be reported (last row). Conversely, version 1 fails to report hits containing mismatches at the ends of the query sequence (third row)

In the original workflow, primer-probe interaction outcomes were derived through manual inspection and interpretation of the AssayBLAST output (Table 1). In contrast, AssayBLAST v2 determines these outcomes automatically by applying the described rule set using strand orientation, spatial proximity, and mismatch counts (Tables 2 and  3). This transition from manual to automated interpretation improves reproducibility, efficiency, and avoids human error, especially when dealing with large and complex assay designs.

A webservice with a similar perspective is Primer-BLAST [15, https://www.ncbi.nlm.nih.gov/tools/primer-blast, last accessed 26/02/2026], which integrates Primer3 [14] for primer design and enables specificity screening of individual primer pairs against a user-selected reference database. In contrast, AssayBLAST is tailored for the evaluation of multiple primer and probe sets within complex assay configurations and provides a comprehensive assessment of their expected amplification outcome across target sequences. The tool further reports unintended or off-target amplification events. As a command-line application, AssayBLAST ensures full user control and maintains complete data sovereignty.

AssayBLAST is not a primer-design tool such as Primer3 or ConsensusPrime [3]. Instead, it should be regarded as a complementary post-design quality-control tool that can be applied after primer and probe selection. Primer3 and Multiple Primer Analyzer (https://www.thermofisher.com, last accessed 19/05/2026) evaluate properties such as melting temperatures, GC content, and primer-primer interactions, but do not perform large-scale screening against sequence databases of interest. AssayBLAST complements these tools by providing sequence-based specificity assessment and target-screening capabilities and can therefore be integrated into existing or newly developed primer-design workflows. AssayBLAST does not model thermodynamic properties such as melting temperatures, secondary structures, primer-dimer formation, or polymerase-specific effects. Consequently, its predictions should be interpreted as sequence-based assessments of assay specificity and amplification compatibility rather than direct predictions of amplification efficiency.

It has been demonstrated that AssayBLAST can accurately predict microarray results. By bridging in silico and experimental validation, AssayBLAST v2 supports more reliable, reproducible, and scalable molecular diagnostic and research workflows.

## Conclusions

AssayBLAST v2 formalizes two critical components that should be integral to any modern primer design pipeline. First, it performs in silico quality control of designed oligonucleotides, including assessment of target binding and strand specificity, primer-probe distance verification, detection of duplicate oligonucleotides, and identification of potential non-specific binding events. Second, it provides a prediction of amplification behavior based on the previously calculated oligonucleotide characteristics, serving as a computational reference for comparison with wet-lab results and subsequent protocol optimization. These advancements establish AssayBLAST v2 as a robust, reproducible, and computationally efficient tool for the in silico evaluation of oligonucleotide-based assays, complementing primer and probe design workflows.

## Data Availability

AssayBLAST is available on GitHub at https://github.com/rnajena/AssayBLAST and archived on Zenodo [7]. Code to reproduce the results is available at https://www.github.com/rnajena/assayblast_paper [6]. The used genomes can be downloaded from NCBI (https://www.ncbi.nlm.nih.gov). The corresponding accession numbers are provided in Eulenfeld (2026) [6].

## Code availability

**Project name** AssayBLAST

**Project homepage** https://github.com/rnajena/AssayBLAST

**Releases** are distributed via PyPI and archived at Zenodo [7]

**Operating systems** Linux, MacOS

**Programming Language** Python

**Other requirements** BLAST, Python 3.11+, rnajena-sugar

**License** MIT

**Restrictions to use by non-academics** MIT license
